# Panomics: New Databases for Advancing Cardiology

**DOI:** 10.3389/fcvm.2021.587768

**Published:** 2021-05-10

**Authors:** Dara Vakili, Dina Radenkovic, Shreya Chawla, Deepak L. Bhatt

**Affiliations:** ^1^Imperial College School of Medicine, Imperial College London, London, United Kingdom; ^2^Hooke London, London, United Kingdom; ^3^Faculty of Life Sciences and Medicine, King's College London, London, United Kingdom; ^4^Brigham and Women's Hospital and Harvard Medical School, Boston, MA, United States

**Keywords:** panomics, systems biology, big data, cardiology, database, proteomics, genomics, methylomics

## Abstract

The multifactorial nature of cardiology makes it challenging to separate noisy signals from confounders and real markers or drivers of disease. Panomics, the combination of various omic methods, provides the deepest insights into the underlying biological mechanisms to develop tools for personalized medicine under a systems biology approach. Questions remain about current findings and anticipated developments of omics. Here, we search for omic databases, investigate the types of data they provide, and give some examples of panomic applications in health care. We identified 104 omic databases, of which 72 met the inclusion criteria: genomic and clinical measurements on a subset of the database population plus one or more omic datasets. Of those, 65 were methylomic, 59 transcriptomic, 41 proteomic, 42 metabolomic, and 22 microbiomic databases. Larger database sample sizes and longer follow-up are often better suited for panomic analyses due to statistical power calculations. They are often more complete, which is important when dealing with large biological variability. Thus, the UK BioBank rises as the most comprehensive panomic resource, at present, but certain study designs may benefit from other databases.

## Introduction

**Figure 1:**
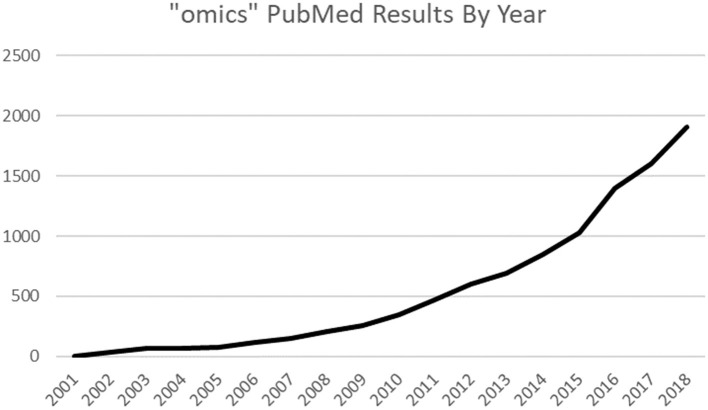
PubMed results trends: “omics” keyword increasing in use.

The biomedical data revolution has begun. The complexity of the cardiovascular system requires huge amounts of data points to provide an effective basis for analysis (1). Modern advances in computational technology and provision of cheaper molecular investigation have allowed fields utilizing giant datasets with the suffix “-omic” (Figure 1) to integrate with research and medicine (2). Panomics is the cross integration of omic measurements taken systematically across samples and can be used for deeper systems biology analyses to determine the origins, relationships, and effects of biological processes (3). Often longitudinal in design, they have broad applicability and potential for use in pharmaceutical research (4). There is growing commercial interest in panomics as, for instance, adding detailed genomic data to an electronic health record increases its value from $130 up to $6,500, setting the value of current UK National Health Service data at $12.5 billion per year (5). Most health data are generated by the academic and public sector, but the health analytics sector 2023 forecast of $22.7 billion (6) is incentivizing private companies. The Global Genomics Group (Table 1), a specialist omic health analytics company, raised millions in funding rounds to generate a commercial omic database.

**Table 1:** The 15 largest databases found using methodology stated in the Methods section.

**Database name**	**Recruitment year**	**Sample size**	**Longitudinal**	**Genome**	**Methylome**	**Transcriptome**	**Metabolome**	**Proteome**	**Phenome**	**Microbiome**	**Intended Speciality**	**Link**
Registre Gironí del Cor (REGICOR)	1978	700,000	Y	Y	Y	Y	Y	Y	Y	N	General	https://www.revespcardiol.org/es-regicor-35-years-of-excellence-articulo-S1885585713002739?redirect=true
UK BioBank	2006	500,000	Y	Y	Y	Y	Y	Y	Y	Y	General	https://www.ukbiobank.ac.uk/
Netherlands Twin Registry	2004	240,000	Y	Y	Y	Y	Y	Y	Y	Y	General	http://www.tweelingenregister.org
LifeLines	2006	167,729	Y	Y	Y	Y	Y	Y	Y	Y	General	http://www.lifelines.nl
Nord-Trøndelag Health Study (The HUNT Study)	1984	120,000	Y	Y	Y	Y	Y	Y	N	N	General	https://www.ntnu.edu/hunt/hunt-samples
FINRISK	1972	101,451	Y	Y	Y	Y	Y	Y	Y	Y	General	https://thl.fi/en/web/thlfi-en/research-and-expertwork/population-studies/the-national-finrisk-study
UK Household Longitudinal Study	2009	100,000	Y	Y	Y	Y	N	N	Y	N	Societal	https://www.understandingsociety.ac.uk/
The Tromsø Study	1974	93,287	Y	Y	N	N	N	Y	N	N	General	https://en.uit.no/om/enhet/artikkel?p_document_id=80172&p_dimension_id=88111
100,000 Genomes Project	2012	70,000	Y	Y	Y	Y	Y	Y	Y	Y	Rare Disease	https://www.genomicsengland.co.uk/about-genomics-england/the-100000-genomes-project/
Estonian Biobank of the Estonian Genome Center, University of Tartu	1999	52,000	Y	Y	Y	Y	Y	Y	Y	N	General	http://www.biobank.ee
INTERVAL	2012	50,000	N	Y	Y	N	N	Y	N	N	Blood Donation	https://www.nature.com/articles/s41586-018-0175-2
National Health and Nutrition Examination Survey (NHANES)	1960	31,126	Y	Y	N	N	Y	Y	Y	N	Nutrition	https://www.cdc.gov/nchs/nhanes/index.htm
EPIC-Norfolk Study	1993	30,000	Y	Y	Y	Y	Y	Y	Y	N	Oncology	http://www.mrc-epid.cam.ac.uk/research/studies/epic-norfolk/
Rotterdam Study (Charge)	1990	19,000	Y	Y	Y	Y	Y	Y	Y	Y	General	http://www.epib.nl/research/ergo.htm
Cooperative Health Research in the Region of Augsburg, Southern Germany (KORA)	1984	18,000	Y	Y	Y	Y	Y	Y	Y	N	General	http://epi.helmholtz-muenchen.de/kora-gen/index_e.php
Multiethnic Cohort (MEC) Study	199 3	215,000	Y	Y	Y	Y	Y	N	Y	Y	Oncology	https://www.uhcancercenter.org/mec
The Singapore Multi-Ethnic Cohort (MEC) study	2004	14,465	Y	Y	Y	Y	Y	N	Y	Y	General	https://pubmed.ncbi.nlm.nih.gov/29452397/
NIHR Cambridge BioResource	2005	17,300	Y	Y	Y	Y	N	N	Y	N	General	https://www.sciencedirect.com/science/article/pii/S0092867416314465
Atherosclerosis Risk in Communities Study (ARIC) (CHARGE)	1987	15,792	Y	Y	Y	Y	Y	Y	Y	N	Cardio	http://www.cscc.unc.edu/aric/
Framingham (CHARGE)	1948	15,447	Y	Y	Y	Y	Y	Y	Y	Y	Cardio	https://www.ncbi.nlm.nih.gov/pmc/articles/PMC4159698/
UK Adult Twin Registry (TwinsUK)	1992	14,274	Y	Y	Y	Y	Y	Y	Y	Y	General Paediatric	http://www.twinsuk.co.uk/
Avon Longitudinal Study of Parents and Children (ALSPAC)	1991	13,988	Y	Y	Y	Y	Y	Y	Y	Y	Paediatric	https://academic.oup.com/ije/article/42/1/97/694445
Fenland Study	2015	12,435	Y	Y	Y	Y	Y	N	Y	N	Endocrine	http://www.mrc-epid.cam.ac.uk/Research/Studies/Fenland/index.html
Northern Finland Birth Cohort 1966	1966	12,058	Y	Y	Y	N	N	Y	Y	Y	General	https://jmg.bmj.com/content/56/9/607
Pain-OMICS	2013	12,000	Y	Y	Y	Y	Y	N	N	N	Pain	https://cordis.europa.eu/project/rcn/110070/factsheet/en
A Large-Scale Schizophrenia Association Study in Sweden	2005	11,850	Y	Y	Y	Y	N	N	N	N	Psychiatry	https://www.nature.com/articles/ng.2742
Metabolic Syndrome in Men (METSIM)	2005	10,197	Y	Y	Y	Y	Y	N	Y	Y	General	https://academic.oup.com/hmg/article/27/10/1830/4939377#118176243
Global Genomics Group (G3) GLOBAL Study	2012	10,000	Y	Y	Y	Y	Y	Y	Y	N	General	https://www.g3therapeutics.com/
COPDGene	2008	10,000	Y	Y	Y	Y	Y	Y	N	N	COPD	http://www.copdgene.org/
Oxford BioBank	1999	8,000	Y	Y	Y	Y	N	N	Y	N	General	https://www.oxfordbiobank.org.uk/
Ontario Familial Colon Cancer Registry (OFCCR)	1998	7,377	Y	Y	Y	N	N	N	N	N	Oncology	https://www.zanecohencentre.com/gi-cancers/ofccr
Multi-Ethnic Study of Atherosclerosis (MESA)	2000	6,814	Y	Y	Y	Y	Y	Y	Y	N	Cardio	https://www.mesa-nhlbi.org/Publications.aspx
National Institute on Aging (NIA) SardiNIA Study	2001	6,148	Y	Y	Y	Y	N	Y	N	Y	Geriatric	http://www.ncbi.nlm.nih.gov/projects/gap/cgi-bin/study.cgi?study_id=phs000338.v1.p1
Corogene	2006	5,809	Y	Y	Y	Y	N	N	Y	N	Cardio	http://ije.oxfordjournals.org/content/early/2011/06/02/ije.dyr090.extract
Age, Gene/Environment Susceptibility-Reykjavik Study (AGES)	2002	5,764	Y	Y	Y	Y	Y	Y	Y	N	Geriatric	http://www.hjarta.is/english/ages
Cardiovascular Risk in Young Finns Study	1980	4,320	Y	Y	Y	Y	Y	Y	Y	N	Cardio	http://youngfinnsstudy.utu.fi/index.html
Study of Health in Pomerania (SHIP)	1997	4,308	Y	Y	Y	Y	Y	Y	N	Y	General	https://pubmed.ncbi.nlm.nih.gov/22736157/
Environment And Genetics in Lung cancer Etiology (EAGLE)	2002	4,000	Y	Y	Y	N	N	N	N	N	Oncology	https://eagle.cancer.gov/background.html
Accessible Resource For Integrated Genomics (ARIES)	2012	3,948	Y	Y	Y	Y	N	N	Y	N	General	http://www.ariesepigenomics.org.uk/
IMT-Progression as Predictors of Vascular Events in a High-Risk European Population (IMPROVE)	2004	3,711	Y	Y	N	Y	N	Y	N	N	Cardio	https://link.springer.com/article/10.1007%2Fs00125-014-3215-y#Sec2
Subpopulations and Intermediate Outcome Measures in COPD (SPIROMICS)	2010	2,981	Y	Y	N	N	N	Y	N	N	COPD	https://www.spiromics.org/spiromics/
Athero-Express Biobank Studies	2002	2,500	Y	Y	Y	Y	Y	Y	N	N	Cardio	https://www.atheroexpress.nl/
Leiden Longievity Study	2002	2,415	N	Y	Y	Y	Y	Y	Y	N	Geriatric	https://www.nature.com/articles/5201508#Sec2
TRAILS (Tracking Adolescents' Individual Lives Survey)	2000	2,230	Y	Y	Y	N	N	N	Y	N	Paediatric	https://www.trails.nl/en
The Orkney Complex Disease Study (ORCADES) (EUROSPAN)	2005	2,080	Y	Y	Y	Y	N	N	Y	N	General	https://www.ed.ac.uk/viking/about-us/orcades
Helsinki Birth Cohort Study	2001	2,003	Y	Y	Y	N	Y	N	N	N	Geriatrics	http://www.ktl.fi/portal/english/research_people_programs/health_promotion_and_chronic_disease_prevention/units/diabetes_unit/idefix_study/
Lothian Birth Cohort 1921 & 1936	1999	1,641	N	Y	Y	Y	Y	Y	Y	N	Cognitive Ageing	https://www.lothianbirthcohort.ed.ac.uk/content/scottish-mental-survey-1947
Conditions Affecting Neurocognitive Development andLearning in Early Childhood Study (CANDLE)	2006	1,503	Y	Y	Y	Y	N	N	Y	Y	Neuro-Paediatric	https://candlestudy.uthsc.edu/
InCHIANTI	1998	1,453	Y	Y	Y	Y	N	Y	Y	N	Geriatric	http://inchiantistudy.net/wp/
The Study Of Colorectal Cancer in Scotland (SOCCS)	1999	1,298	Y	Y	Y	Y	Y	N	Y	N	Oncology	https://www.ed.ac.uk/usher/molecular-epidemiology/our-studies/the-study-colorectal-cancer
Cardiovascular Health Study (CHARGE)	1989	1,250	N	Y	Y	Y	N	N	N	N	Cardio	https://www.ncbi.nlm.nih.gov/projects/gap/cgi-bin/study.cgi?study_id=phs000287.v7.p1
Growing Up in Singapore Towards healthy Outcomes (GUSTO)	2009	1,176	Y	Y	Y	Y	Y	Y	Y	Y	Paediatric Metabolism	https://academic.oup.com/ije/article/43/5/1401/695117
Northern Sweden Population Health Study (EUROSPAN)	2006	1,069	Y	Y	Y	Y	Y	Y	Y	N	General	http://eurospan.gen-info.hr/partners.html
HELMi (Health and Early Life Microbiota)	2016	1,055	Y	Y	N	N	N	N	N	Y	Microbiome & Paediatrics	https://bmjopen.bmj.com/content/9/6/e028500.long
Prospective Investigation of the Vasculature in Uppsala Seniors (PIVUS)	2001	1,016	Y	Y	Y	N	N	N	Y	N	Cardio	https://bmcmedgenomics.biomedcentral.com/articles/10.1186/s12920-016-0235-0
VIS (part of EUROSPAN)	2003	1,008	Y	Y	Y	Y	N	N	Y	N	General	http://eurospan.gen-info.hr/partners.html
Milieu Intérieur cohort	2012	1,000	N	Y	Y	Y	Y	Y	Y	Y	Immunology	https://www.nature.com/articles/s41590-018-0049-7
GOLDN study		968	Y	Y	Y	Y	N	N	N	N	Cardio	https://www.ncbi.nlm.nih.gov/pmc/articles/PMC2952572/
Brisbane systems genetics study (BSGS)		962	Y	Y	Y	Y	N	N	Y	N	Complex Disease	https://journals.plos.org/plosone/article?id=10.1371/journal.pone.0035430
KORCULA (Part of EUROSPAN)	1999	944	N	Y	Y	Y	Y	N	N	N	Cardio	https://www.ncbi.nlm.nih.gov/pmc/articles/PMC2657564/
Diet, Obesity, and Genes (DIOGenes)	2005	932	N	Y	Y	Y	N	Y	N	N	Obesity	https://www.nature.com/articles/s41467-017-02182-z
Center for the Health Assessment of Mothers and Children of Salinas (CHAMACOS) cohort	1999	800	Y	Y	Y	Y	Y	N	Y	Y	Farm exposure eg pesticides	https://www.ncbi.nlm.nih.gov/pmc/articles/PMC6444381/
Alzheimer's Disease Neuroimaging Initiative (ADNI)	2004	800	Y	Y	Y	Y	Y	Y	N	Y	Alzheimer's	http://adni.loni.usc.edu/
AddNeuroMed		700	Y	Y	Y	Y	N	Y	N	N	Alzheimer's	https://consortiapedia.fastercures.org/consortia/anm/
Emory Twin Study (ETS)	1946	614	Y	Y	Y	N	Y	N	Y	N	General	https://link.springer.com/article/10.1186/s13148-016-0189-2
Cross-sectional analyses conducted in the Cohort on Diabetes and Atherosclerosis Maastricht (CODAM)	1999	574	N	Y	Y	Y	Y	N	N	N	Cardio	https://www.sciencedirect.com/science/article/pii/S0009898103005308#aep-section-id12
Qatar Metabolomics Study on Diabetes (QMDiab)	2012	388	Y	Y	Y	Y	Y	Y	Y	N	Endocrine	https://www.ncbi.nlm.nih.gov/pmc/articles/PMC5886112/
Human Microbiome Project	2008	300	Y	Y	N	Y	Y	Y	N	Y	General	https://hmpdacc.org/ihmp/overview/data-model.php
Human Adult Cerebellum Samples	-	153	N	Y	Y	Y	N	N	N	N	Psychiatry	https://www.sciencedirect.com/science/article/pii/S000292971000087X
Human Adult Brain Samples-Cerebellum, Frontal Cortex, Caudal Pons and Temporal Cortex	-	150	N	Y	Y	Y	N	N	N	N	Neurology	https://journals.plos.org/plosgenetics/article?id=10.1371/journal.pgen.1000952
Whole blood from healthy individuals of Dutch origin	-	148	N	Y	Y	Y	N	N	N	N	General	https://bmcgenomics.biomedcentral.com/articles/10.1186/1471-2164-13-636#Sec13
Japanese Study on CSF Proteomic Profile		133	N	Y	N	N	N	Y	N	N	Neuro	https://academic.oup.com/hmg/article/26/1/44/2595397

*Y refers to the given data type being found, and N means it was not found*.

*Recruitment year: The year when participants were recruited, not the year of any retrospective historical event. Sample size: Total database sample size was chosen because sub-population omic data may desirably characterize the overall sample. Longitudinal: Longitudinal study design. Genome: Availability of whole-genome data. Methylome: Deoxyribonucleic acid (DNA) methylation data available as methylation arrays or deep sequencing. Transcriptome: Single-base ribonucleic acid (RNA) reads or mRNA expression data obtained via cRNA microarray chips. Metabolome and Proteome: Appropriate separation and detection methods, such as gas chromatography coupled with mass spectrometry or nuclear magnetic resonance. Broad coverage immuno-assays were also acceptable. Routine clinical blood results do not constitute metabolomics data. Phenome: Traits in individuals not recorded for clinical purposes or clinical techniques, for example, a heart rate monitor to characterize an individual's daily exercise rate. Microbiome: Characterization of participants' microbiomes either with genomic sequencing or growth characterization*.

Cardiovascular risk scoring models consider clinical parameters, such as age, sex, past medical, and drug history. They efficiently assess cardiovascular disease risk in patients who may benefit from prophylactic or active treatment (7). These models brought modest reductions in cardiovascular morbidity rates (8), but utilizing omic data can improve them (9) with features, such as polygenetic risk scores (10).

**Figure 2:**
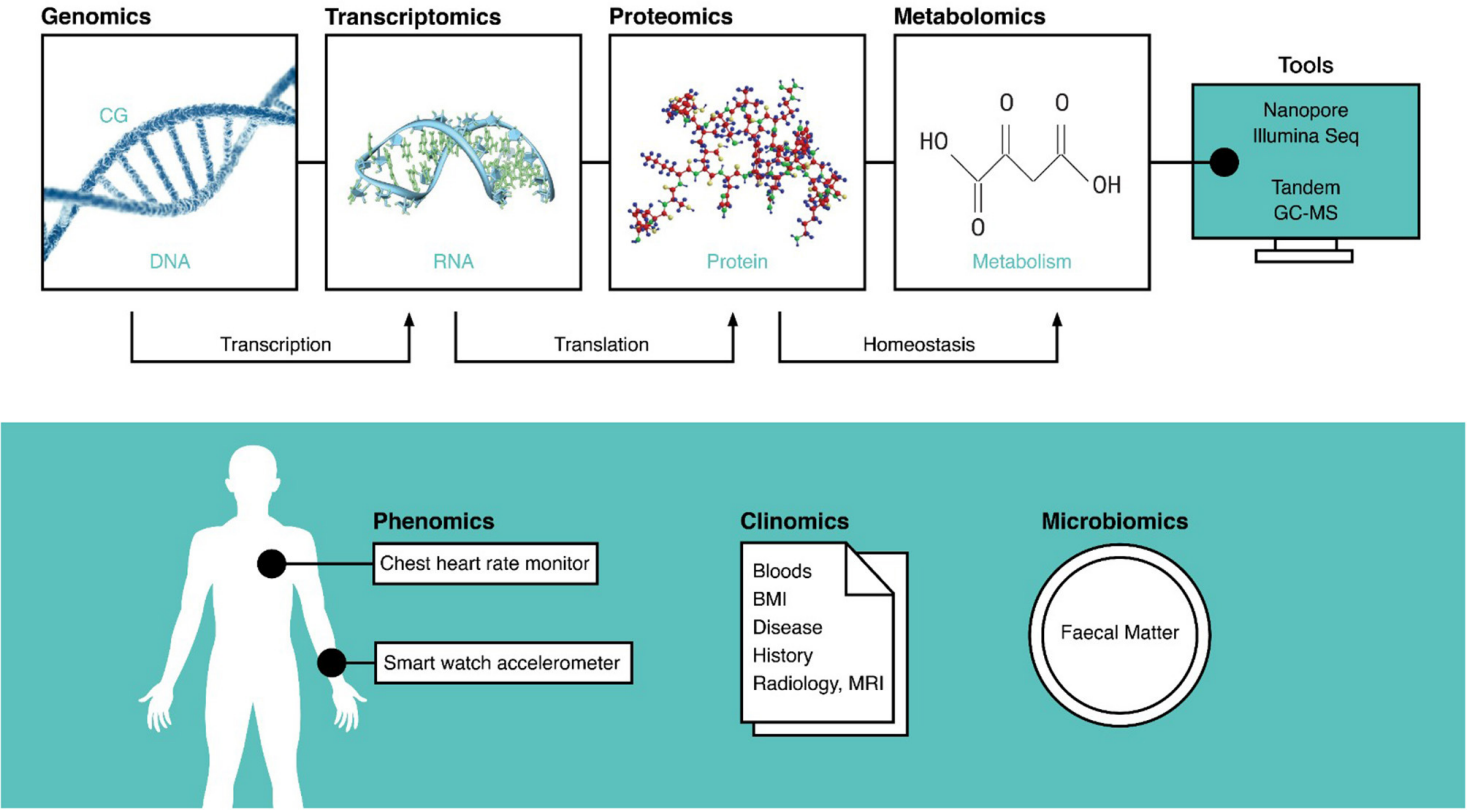
A summary of all the omic data types, the tools used to record them, and the molecular processes they inform. The techniques on top are often invasive and require tissue samples, but those on the bottom are extrinsic and can be measured non-invasively. DNA, deoxyribonucleic acid; CG, cytosine guanine methylation site; RNA, ribonucleic acid; MRI, magnetic resonance imaging; BMI, body mass index; GC-MS, gas chromatography–mass spectroscopy.

Omic databases are particularly useful when investigating factors affected by large biological variation, but exponentially larger samples sizes are needed when multiple forms of omic data are used (11). After data generation, descriptive statistics summarize the data with averages and frequencies. Predictive analytics using artificial intelligence read omic data as a training model to make future predictions for individuals. Prescriptive analytics are most commonly used in medical studies that cluster traits in a population, such as a symptom, to a pattern, such as the differential splicing of a gene (12, 13). The data types often found in omic databases are summarized in Figure 2.

We reviewed which databases existed for panomic analyses, the data types available, and how best they can be utilized. Skepticism remains about their utility, partly because some direct-to-consumer analyses passed the fees of panomic data generation to consumers. Sometimes, this outweighed the gain of personalized insights on health optimization information, such as dietary and exercise recommendations, that were known at the time (14).

## Methods

Population-based databases associated with omic data were found using the following omic keywords: “GWAS,” “Genomic,” “Phenomic,” “Clinomic,” “Proteomic,” “Metabolomic,” “Methylomic,” and “Transcriptomic” on PubMed/Medline and internet searches for existing database websites and gene mutation directories. Individual publications were traced backwards, and authors were contacted for missing data from the Table 1. Databases were included if they contained genomic data plus one or more of the above omic datasets on participants and full clinical information. Study methods were checked for omic data collection techniques, such as mass spectroscopy, Illumina sequencing chips, and data logging wearables. Selected key publications were summarized.

The data mining exercise identified 104, of which 72 met the selection criteria by having sufficient omic and clinical data on study participants. Out of the 72 studies, only one was commercial. The 15 with the largest sample size and fully complete are selected for Table 1.

A “Y” in Table 1 states that omic data were found with enough evidence. An “N” states that evidence for that data type was not found; some reasons are discussed below.

## Results

Overall, 73 omic databases matching the inclusion criteria were identified. Sixty-five databases included methylomic data, 59 included transcriptomics, 41 included proteomics, 42 included metabolomics, 45 included phenomics, and 20 included microbiomics.

### Genomics

Genetics concerns the genome at the base pair level looking at the basic structure of the cellular DNA. Often, genetic studies focus on greatest diversity mediated by single-nucleotide polymorphism (SNP), which is a single base pair alteration resulting from mutative mechanisms. The severity depends on the site and downstream translation of the mutation.

Understanding SNP pathogenicity may help identify targets for personalized medicine. A recent randomized controlled trial investigated replacing clopidogrel, a common antiplatelet activated by cytochrome *2C19*, with ticagrelor or prasugrel in carriers of defective cytochrome *2C19* alleles (15, 16). Genomic studies uncovered loss of function *PCSK9* mutations driving increased low-density lipoprotein cholesterol (LDL-C) receptor recycling. Three subsequent clinical trials of *PCSK9* monoclonal antibody inhibitors showed reduced major adverse cardiovascular events and 60% reductions in plasma LDL-C (17).

Whole-genome sequencing allows computational algorithms to compare all genetic alterations across large samples to isolate patterns related to qualitative traits. Currently, three techniques are popular for genomic analyses. Microarrays are bead chips with well-defined protocols for sample hybridization, which explore many sites in the genome for predetermined sequences. Specialized chips are available, such as genotyping microarrays that screen for known congenital abnormalities (18, 19). The limitations of microarrays can be circumvented by high-throughput sequencing, when sequence reads are produced concurrently in parallel. Illumina sequencing cuts DNA into snippets typically shorter than 600 base pairs and generates short reads, which are assembled against a reference genome giving the full sequence (19). Larger DNA alterations, such as structural variants and repetitive regions cause an ambiguous short-read assembly, and an estimated 15–20% of genetic material including the chromosomal telomeres are missed; hence, long reads are becoming more popular in the comprehensive research testing setting (20). Nanopore, a single-molecule real-time sequencer, allows a single genetic sequence to pass through a pore reading up to ~2,000,000 base pairs. Compared with PacBio's long read method, it offers significantly longer read lengths, higher read accuracy, and lower cost. Each Nanopore detector reads a single strand at a time, making it the least high-throughput method (21, 22).

Various commercial direct-to-consumer genomic tests, summarized in Table 2, are marketed to the public as tools for inferring family ancestries, providing insights into health and well-being, genetic counseling and family planning, drug response analysis, dietary and fitness optimization, and paternity testing, among other uses. A common model they use is a one-time test kit purchase wherein the consumers are given their analyses, but consumers need further membership plans to receive updates from future genomic discoveries on their DNA. The emergence of direct-to-consumer testing kits has been controversial (31) because genomes associated with medical data hold intrinsic fiscal value of up to $6,500 (5), but companies typically charge consumers for sample processing fees. For example, 23andme (Table 2) asks customers if they wish for their data to be used in drug development for which 80% consent to; thus, their data were used to begin drug development on a bispecific monoclonal antibody that blocks IL-36 (32). Questions as to whether the consumer or company owns the data, whether it is ethical for the consumer to waive ownership of their data including their right to any fiscal returns for future innovations, how access to genomic data should be managed, and finally how much education consumers should receive before trading their genetic data have not yet been answered. It is possible that the use of private encryption keys, similar to those used in blockchain technologies (33), may sufficiently control access and protect consumers.

**Table 2:** DNA testing kits available direct for consumer use and for scientific studies.

**Method**	**Technology**	**Price**
100 × Whole Genome Sequencing DNA Test [Nebula Genomics, USA (23)]	Whole-genome sequencing	$3,500
Circle Premium [Prenetics, Hong Kong (24)]^*^	Whole exome sequencing	$629
Health + Ancestry Service [23andme, USA (25)]	Illumina Global Screening Array chip	$199
Ancestry and Well-being Kit [LivingDNA, UK (26)]	Thermo Fisher Scientific Affymetrix chip	$179
TellMeGen DNA Kit [TellmeGen, Spain (27)]	Illumina Global Screening Array chip	$139
AncestryDNA + Traits [Ancestry, USA (28)]	Illumina Omniexpress-24 chip	$119
MyHeritage DNA Kit [MyHeritage, Israel (29)]	Illumina OmniExpress-24 chip	$79

* *Prenetics, Hong Kong has acquired circle DNA (24) and DNAfit (30)*.

Genome-wide association studies (GWAS) found loci associated with elevated LDL-C and incidence of coronary artery disease (CAD) (34). This led to the generation of polygenetic risk scores by identifying associations between traits in a training sample, and single or combinations of genetic markers that present little significance solely in association studies (35). Polygenetic risk scores made from UK BioBank participants (Table 1) identified that 8% of the population had a 3-fold risk of developing CAD, of which most displayed healthy blood profiles otherwise denoting undetectable risk (36). The metaGRS risk prediction model (10) found that UK BioBank individuals in the two top deciles had a hazard ratio of 4.17 as compared with those in the bottom two deciles. High CAD prevalence is increasing due to trends in developing countries (37), reflecting that a large number of people globally are unaware of their CAD risk and perhaps action. Additional UK BioBank data found two loci strongly associated in diabetes patients (38), highlighting that genomic screening could find implications from related conditions. Genetically susceptible patients may have a 46% risk reduction of coronary artery events, who overall have a 91% relative risk at the top quintile compared with the lowest quintile in one study (39).

### Methylomics

Methylation is a dynamic process whereby methyl transferases methylate CpG dinucleotides, repressing DNA transcription without altering base pairs. Methylomics is relatively new and measures epigenetic DNA methylation (40) for assessing carcinogenesis, gene silencing, and aging, among others. Modern personalized age clocks consider methylation patterns to estimate chronological and phenotypic age corresponding to estimated disease mortality (41) and to discern the age of developmental tissue (42) and the time remaining before developing age-related illnesses, such as cardiovascular diseases (43).

Adoption of methylomics into medical data collection is slowed by a lack of cheap, reliable, and interpretable tests. Sixty-five databases in Table 1 include, at the least, a rudimentary level of methylomic analysis. A benefit of in-house DNA sequencing or microarrays is that it gives total flexibility over which tissues and cells to isolate DNA.

Illumina's Epic DNA methylation microarray kit samples 850,000 CpG known sites (44); however, this does not account for the total biological variability of DNA methylation. Full sequencing using Illumina DNAseq technology or Nugen's TrueMethyl oxBS-Seq Module (45, 46) introduces great cost because next-generation sequencers cannot detect methyl-cytosines, so a whole-genome read is compared with an additional read generated by bisulfite conversion (47), whereby cytosines are converted into uracil and then thymine, but methyl-cytosines remain unchanged. This is also known as whole-genome bisulfite sequencing.

Three commercial kits are summarized in Table 3, which sample different numbers and locations of CpG sites. The DNAge test (Zymo Research, USA) (52) reports methylomic age, estimates chronological age, provides summary statistics and graphics for integration into clinical studies, and estimates chronological ages of samples; however, it lacks more detailed information. Details of the algorithmic methods of commercial tests are often not publicly available.

**Table 3:** Three DNA methylation testing kits available direct for consumer use and for scientific studies.

**Method**	**Tissue sampled**	**Price**
Index [Elysium Health, USA (48)]	Saliva	$499 (49)
DNAge [Zymo Research, USA (50)]	Blood or Urine	$299 per consumer test
Chronomics [Chronomics, UK (51)]	Saliva	£900–1,499

Leukocyte DNA methylation is useful in determining links between smoking and pathogenesis (53). A Euro-American meta-analysis involving 11,461 participants' leukocytes found 52 associative and two causal CpG sites for CAD development affecting genes involved in calcium regulation and kidney function (54). Findings, such as this may serve as a tool to optimize risk predictions in smokers for developing CAD and to unveil more information into the molecular and cellular mechanisms driving pathogenicity. If repeated, this analysis may better address cell type variability if leukocyte sub-type data were available (55, 56) or if a single-cell analysis was used. Additionally, the use of panomics has epigenetic regulation and pathology. A UK Household Longitudinal Study (Table 1) made an online searchable database of 12,689,548 methylation quantitative trait loci (QTLs) associated with 2,907,234 genetic variants and 93,268 methylation sites in 1,193 individuals' blood samples. These were associated to 60 human traits including pleiotropic mapping of complex traits and changes in gene expression for 1,702 genes (57).

### Transcriptome

Gene expression can be measured with transcriptomics, which reads cRNA, processed from mRNA, and is useful for assessing relationships between regulatory elements and phenotypes (58). For example, *PCSK9* mRNA was degraded with a single dose of RNA interfering Inclisiran, reducing LDL-C by 57% for 240 days in phase II trials (59, 60) which may be a cheaper alternative to evolocumab (61).

Transcriptomics are measured with RNA sequencing or microarrays for predetermined mRNA sequences. Conversely to genomics, RNA isolation and amplification kits are used, and different algorithms ensure read alignment and quality control (58). A total of 59 databases were found to include transcriptomic data, and most used microarrays.

Links between anomalous cardiac QRS complexes in individuals who have higher differential expression and methylation across 52 genetic loci have been identified (62). Transcriptomics can also be used for assessing alternative and differential splicing events (63). A 97-nucleotide splice insert in the *LDL-R* transcript caused familial hypercholesteremia in participants who otherwise did not carry any known *LDL-R* mutations (64).

### Proteomics

Forty-one databases included proteomic data. Proteomics analyze the structure of isolated proteins and quantify expression (65) with gas or liquid chromatography coupled with tandem mass spectroscopy as a gold standard, or cheaper methods, such as matrix-assisted laser desorption/ionization–time of flight. Bioinformatics process data and model protein–protein interactions and drug targets, among others (66). It is a specialist technique carried out less often, and its applicability to general clinical practice is unknown.

The downstream effects of most discovered splicing events are unknown, and only one software (67) can predict novel events solely using transcriptomic data. A study in pre-print amalgamated data from existing transcriptomic and proteomic databases and found 253 novel splice peptides in 212 genes undocumented in existing annotations (68).

The Framingham Heart Study (Table 1) facilitated extensive proteomic studies. Plasma proteins of 2,100 participants were examined against the net Framingham cardiovascular disease risk score, identifying 161 novel genetic variants that account for 66% of plasma protein concentration variation in cardiovascular disease participants (69). A total of 6,861 participants' plasma were examined, finding 16,000 protein QTLs mapped against 71 cardiovascular disease proteins with functional relevance to CAD and eight as useful predictors of new-onset cardiovascular disease events (70). The expression of 85 protein biomarkers previously associated with CAD in genomic studies was measured to fine-tune hazard ratios for cardiovascular outcomes (71).

### Metabolomics

Protein disturbances can alter metabolites that change one's metabolomic profile (72), which may be retrospectively investigated to identify protein disturbances (73). Analyzers used in proteomics are used with emphasis on metabolite isolation. Targeted metabolomics focus on predetermined metabolites expected to react with environmental changes. Untargeted metabolomics attempt to provide full coverage of all metabolites but are more resource intensive (74). Forty-two databases on Table 1 have metabolomic data.

A total of 105 metabolites were significantly altered in Chinese patients with CAD, including palmitic acid, linoleic acid, and phosphatidylglycerol, which have variable associations with CAD (75).

Twins UK (Table 1) facilitated advances on human metabolomics. A total of 145 genetic loci related to levels of 400 plasma metabolites where characterized against gene expression and heritable loci associated with complex disease phenotypes. Mapping loci and biochemical pathways may assist drug and biomarker discovery (76). Combining this with other databases including EPIC-Norfolk (Table 1), a meta-analysis in 80,003 participants discovered 22 genetic variants associated with circulating glycine, further suggesting that glycine is protective in CAD (77).

### Phenomics

Phenomics consider phenotypes, information on observable traits, and morphology, such as dieting, exercise, and sleep from wearables (Figure 2). Overlaps with clinical data can be discerned via the methods. Cardiopulmonary exercise data are interventional and therefore clinical, whereas daily heart rate data collected with a wearable are phenotypic (78). Smart watches and phones enable development of mobile health platforms (79) that conveniently collect daily physical exertion, geolocation, and dietary data, among others. While simple and user friendly, wearables, such as watches measuring heart rates have low accuracy (80, 81).

Current apps have focused on health optimization, but medical interventions are emerging; for example, the iHeart study evaluates whether participants' atrial fibrillation outcomes can be improved using “behavior-altering motivational” messages based on an iPhone-connected ECG monitor (82).

A total of 103,578 UK BioBank participants aged between 45 and 79 years had wrist-worn accelerometers that record daily physical activities (83) and automatically categorize these activities into groups, such as cycling or walking and record sleep cycle stage (84). Long-term physical activity is pivotal in cardiovascular health and recovery (85), and these data could improve risk models. Forty-five databases in the Table 1 include phenotypic data.

### Microbiomics

The accessory genome is larger than the human genome (86). Microbiomics use omics to characterize resident microbiota commonly in the gut, skin, and lungs. Twenty databases included microbiomic profiles on the Table 1.

Some private biotechnology companies use microbiomics to personalize diets. Zoe, UK, found differences in obesity, diabetes, and heart disease risk in identical twins with dissimilar microbiomes. Their trial had success in predicting more suitable dietary guidance (87). Viome, USA, sells $129 consumer kits and offer dietary advice via smart phones (88). Groups at the Weizmann Institute are using post-meal glucose spikes captured by continuous glucose-monitoring devices (89).

A study combining metabolomic and microbiomic data of 617 middle-aged women found that less diverse microbiomes were correlated with higher arterial stiffness, greater visceral fat, and increased insulin resistance (90). Bacterial genes associated with development of atherosclerotic disease (91) and increased levels of trimethylamine N-oxide were discovered (92). This information may help to improve risk models or to modulate bacterial communities for better health.

LifeLines (Table 1) include fecal sample banking. In 2019, highlighted studies discovered gut bacterial species associated with increased incidence of depression (93), and causal effects of butyrate-producing bacteria on metabolic traits confirmed by measuring glucose-stimulated insulin response and fecal short-chain fatty acids (94) and using bacterial species associated with obesity and poor lipidemia to improve cardiovascular risk models (89, 95).

#### Analytical Methods

Analyzing omic data is computationally intensive and is often carried out using powerful computers, known as clusters, placed behind the owner institution's firewall. Otherwise, institutions or researchers granted access can download data to their own secure clusters. Initially, bioinformatics approaches relied heavily on experimentally validated domain expertise to make knowledge-driven inferences on specific pathways or genes. Now, the generation of panomic databases exists alongside a rich selection of data-driven methods for research and discovery, each with their own technical advantages and limitations. The selection of the best combination of omic data integration tools is dependent on the use case but is outside the scope of this study. Most can be classified as multivariate, fusion, Bayesian, network, correlation, and similarity (96).

Multivariate Mendelian randomization (97) is a technique used to discern causality in observational studies between modifiable lifestyle risk factors and disease while minimizing the effects of confounders. For example, two panels of ~350 SNPs were selected from 2,436,300 SNPs identified in GWAS data. Using these SNPs as instrumental variables, LDL-C was identified as a causal driver of CAD, but HDL-C was protective, whereas risk from plasma triglycerides was dependent on LDL-C levels (98).

Often data can be missing for a variety of reasons; for example, methylation microarray chips only sample a limited number of CpG sites on the genome, as stated previously. Imputation is a technique where statistical inferences, assuming similar patterns are represented across samples, can be made on unobserved data points, such as CpG sites. The mixture regression model (99) is one imputation method that has been demonstrated to recover methylation data, achieving a correlation rate of 80% when up to 80% of the methylation data points have been deleted. Combining whole-genome bisulfite sequencing data from a subsample with microarray data of the wider sample as an input for the algorithm increases the prediction scope, while the cost of analysis is reduced.

Network analyses are often used to combine findings between different sets of omic data. Simplistically, a network is a set of nodes that represent variables, and the relationships between them, known as edges, can be explored. Methylomic, metabolomic, and proteomic data were combined to form a multi-layered network whereby the omic data sources were matched with sources of healthy and calcified aortic valves. The novel networks in this study found associations between amyloid deposits on aortic valves in Alzheimer's patients and highlighted associated genes to the valve spongiosa layer, which has previously not been central to calcific aortic valvular disease research (100). Network methods, specifically deep neural networks, attracted the public eye after Google DeepMind's AlphaFold 2 model predicted protein folded structures using only the amino-acid sequence with near-identical performance as gold standard experimental methods, such as cryo-electron microscopy (101, 102).

## Discussion

Seventy-three databases were found containing omic data across a range of countries, specialties, and study designs. All databases include genomic and clinical data, as this is a quintessential reference for any health panomic analysis and most are a cohort or retrospective-cohort design. Table 1 shows that databases with larger sample sizes cover more omic data types, as the techniques and expertise required for each omic technique are resource intensive and are often best facilitated with larger databases.

Initial studies on Mendelian disease identified common disease-causing variants within DNA coding regions (103). Early GWAS are built on these and identified genetic variants associated with disease, which is useful for risk prediction models (104). Deeper and cheaper molecular investigation techniques enable inclusion of mRNA sequencing and DNA methylation to measure the effect of regulatory elements and their contributions to Mendelian and complex disease (105). Variants associated with biological traits that underlie increased disease risk have been explored less (106). Panomics addresses this by amalgamating omics with phenotypic and clinical data to deluge interactions between biological mechanisms and pathophysiology.

The following databases from Table 1 are recommended for panomic health data analysis, as they have large sample sizes, are longitudinal, and include a wide breadth of omic data. The UK BioBank has a larger sample size and detailed clinical and phenotypic data systematically organized that are available for research access. It has contributed to large numbers of epidemiological studies, risk scoring, and prediction models and has helped characterize associative and causal factors linked with life-threatening illnesses including cancer, cardiovascular disease, dementia, and diabetes. The Netherlands Twin Registry (Table 1) and TwinsUK follow suit with smaller sample size but are particularly useful for quantifying the effect of genetic and environmental factors behind human traits. The LifeLines study follows up participants across three generations for at least 30 years to study hereditary traits and aging. The 100,000 Genomes Project is useful for rare diseases or rare disease models. The Nord-Trøndelag Health study and FINRISK (Table 1) started in 1984 and 1972 were not originally dedicated to omics but have clinical data available over longer follow-up periods.

Omic databases have ethnic shift toward White European ancestries, limiting their clinical use in ethnically diverse populations (107, 108). Of the databases identified, few were generated in Asia, one (Table 1) was generated on Middle Easterners (109), and none was generated in Africa, although efforts have been made to include other ethnicities in Northern American and European databases (110) (Table 1).

Databases using detailed public-facing websites summarizing the types of data available were more easily identifiable. Most websites either did not include the types of measurements carried out or have not been updated. Databases with complex or long names or non-unique names had search results muddied with irrelevant results. Although in this review various panomic studies have been identified, the availability of the data strongly depends on local governance and privacy laws, except for dedicated open-access or requested-access databases, such as the UK BioBank. This review highlights the need for a database of databases for which principal investigators register their studies and include conclusive information for the academic community.

## Author Contributions

DV wrote the bulk of the text, performed the literature search and review under guidance from DR, who also reviewed the text. SC reviewed the text. DB was the senior supervisor for this work and reviewed the text. All authors contributed to the article and approved the submitted version.

## Conflict of Interest

DV is a founding member and product manager of Salutare Group Ltd, London, UK. Salutare was in no way involved in the production of this work. DR is the Chief Science Officer at Health Longevity Performance Optimisation Institute (HLPO.Life), London, UK. DB discloses the following relationships—Advisory Board: Cardax, CellProthera, Cereno Scientific, Elsevier Practice Update Cardiology, Level Ex, Medscape Cardiology, PhaseBio, PLx Pharma, Regado Biosciences; Board of Directors: Boston VA Research Institute, Society of Cardiovascular Patient Care, TobeSoft; Chair: American Heart Association Quality Oversight Committee; Data Monitoring Committees: Baim Institute for Clinical Research (formerly Harvard Clinical Research Institute, for the PORTICO trial, funded by St. Jude Medical, now Abbott), Cleveland Clinic (including for the ExCEED trial, funded by Edwards), Contego Medical (Chair, PERFORMANCE 2), Duke Clinical Research Institute, Mayo Clinic, Mount Sinai School of Medicine (for the ENVISAGE trial, funded by Daiichi Sankyo), Population Health Research Institute; Honoraria: American College of Cardiology (Senior Associate Editor, Clinical Trials and News, ACC.org; Vice-Chair, ACC Accreditation Committee), Baim Institute for Clinical Research (formerly Harvard Clinical Research Institute; RE-DUAL PCI clinical trial steering committee funded by Boehringer Ingelheim; AEGIS-II executive committee funded by CSL Behring), Belvoir Publications (Editor in Chief, Harvard Heart Letter), Duke Clinical Research Institute (clinical trial steering committees, including for the PRONOUNCE trial, funded by Ferring Pharmaceuticals), HMP Global (Editor in Chief, *Journal of Invasive Cardiology*), *Journal of the American College of Cardiology* (Guest Editor; Associate Editor), K2P (Co-Chair, interdisciplinary curriculum), Level Ex, Medtelligence/ReachMD (CME steering committees), MJH Life Sciences, Population Health Research Institute (for the COMPASS operations committee, publications committee, steering committee, and USA national co-leader, funded by Bayer), Slack Publications (Chief Medical Editor, Cardiology Today's Intervention), Society of Cardiovascular Patient Care (Secretary/Treasurer), WebMD (CME steering committees); Other: Clinical Cardiology (Deputy Editor), NCDR-ACTION Registry Steering Committee (Chair), VA CART Research and Publications Committee (Chair); Research Funding: Abbott, Afimmune, Amarin, Amgen, AstraZeneca, Bayer, Boehringer Ingelheim, Bristol-Myers Squibb, Cardax, Chiesi, CSL Behring, Eisai, Ethicon, Ferring Pharmaceuticals, Forest Laboratories, Fractyl, Idorsia, Ironwood, Ischemix, Lexicon, Lilly, Medtronic, Pfizer, PhaseBio, PLx Pharma, Regeneron, Roche, Sanofi Aventis, Synaptic, The Medicines Company; Royalties: Elsevier (Editor, Cardiovascular Intervention: A Companion to Braunwald's Heart Disease); Site Co-Investigator: Biotronik, Boston Scientific, CSI, St. Jude Medical (now Abbott), Svelte; Trustee: American College of Cardiology; Unfunded Research: FlowCo, Merck, Novo Nordisk, Takeda. The remaining author declares that the research was conducted in the absence of any commercial or financial relationships that could be construed as a potential conflict of interest.
